# Pre-operative management of fracture blisters: a systematic review

**DOI:** 10.1530/EOR-2024-0074

**Published:** 2025-03-03

**Authors:** Ishtar Redman, Kapil Sugand, Aashtad Daruwalla, Andrew Clark

**Affiliations:** ^1^ Department of Trauma and Orthopaedic Surgery, University College London Hospitals NHS Foundation Trust, London, UK; ^2^ Department of Trauma and Orthopaedic Surgery, Royal National Orthopaedic Hospital, Stanmore, UK; ^3^ Department of Surgery & Cancer, Imperial College, London, UK; ^4^ Department of Trauma and Orthopaedic Surgery, Queen Elizabeth Hospital, Gateshead Health NHS Foundation Trust, Gateshead, UK

**Keywords:** fracture blisters, bone blisters, pre-operative management, deroof, aspiration, silver sulfadiazene, serous blisters, haemorrhagic blisters

## Abstract

**Purpose:**

- The pre-operative management of fracture blisters is an area of uncertainty within trauma and orthopaedic surgeries. Management strategies vary significantly between and within orthopaedic departments across the United Kingdom. The purpose of this systematic review was to comprehensively appraise and synthesize the existing literature pertaining to this topic, highlighting current practices and areas for ongoing research.

**Methods:**

- Extensive electronic literature searches were performed on PubMed/MEDLINE (January 1946–May 2024), Embase (January 1974–May 2024) and Cochrane library (January 1933–May 2024) databases. The search terms were as follows: (fracture blister OR bone blister*) AND (dress* OR drain* OR aspirat* OR deroof* OR manage*). These keywords were searched in the subject headings, in title and in abstract.

**Results:**

- The results of the search methodology revealed five articles, which represented the best evidence to the clinical question. These papers reported on rates of wound healing and post-operative infection, time to surgical readiness and treatment costs, following varying treatment modalities in 1162 patients. The authors, publication dates, countries, patient groups, study outcomes and results of these papers are tabulated in Supplementary Table 1.

**Conclusion:**

- Fracture blisters pose a significant challenge in clinical practice, leading to delays in surgery, suboptimal surgical approaches and complications in wound healing post-operatively. Currently, there is no consensus describing the optimal management of these blisters. This review challenges the conventional belief that fracture blisters are sterile, highlighting that the application of topical agents to the deroofed blister bed may expedite surgical readiness.

## Introduction

Successful management of any fracture requires careful consideration of soft tissue trauma in addition to the underlying bony injury, especially if there is an intention to operate. Fracture blisters are relatively uncommon, estimated to occur in 2.9% of all acute fractures requiring hospitalization (1). These blisters often appear in locations with a paucity of subcutaneous tissue, such as the foot, ankle, wrist, leg and elbow where skin is tightly adhered to the underlying bone.

### Clinical appearances

Clinically, these blisters appear as tense, fluid-filled bullae and can be clear (serous), blood-filled (haemorrhagic) or a combination of both. They often traverse the skin over the underlying fracture, an area termed the ‘zone of injury’. Histologically, the blister subtypes differ by the depth and extent of injury to the dermal–epidermal junction, with clear fluid-filled fracture blisters demonstrating intra-epidermal cleavage above the stratum granulosum layer, retaining a partial epidermal and dermal base (2). In contrast, haemorrhagic fracture blisters demonstrate complete separation of the epidermal layer from the underlying dermis, signifying a greater injury both clinically and histologically (2, 3).

### Biomechanics

This theory is supported by the biomechanical study conducted by Giordano and coworkers (3), in which 60 cadaveric ankle-skin specimens were subjected to uniaxial strain, aimed at reproducing the dermal–epidermal injury purported as the mechanism of fracture blister formation. The authors reported that specimens subject to a uniaxial strain of 152% demonstrated complete separation of the epidermis from the dermis, a similar histological picture to that of clear fluid-filled fracture blisters. Specimens strained to 167% demonstrated a histological picture analogous to that of haemorrhagic fracture blisters with complete separation of the dermal and epidermal layers (4).

These blisters often indicate a significant underlying tissue trauma and typically occur in a 6- to 72-h window post-injury. Although fracture blisters can appear following any fracture with associated soft tissue injury, the most common anatomical areas for the development of these blisters are the ankle, hindfoot and proximal tibia (5). In addition, some fracture patterns are associated with higher rates of blister formation particularly correlated with high-energy and multi-fragmentary injuries, such as tibial pilon and tongue-type calcaneal fractures (5, 6). Despite the pathogenesis of these blisters being well described, review of the literature reveals no consensus on pre-operative fracture blister management. Furthermore, current national guidelines from the British Orthopaedic Association (BOAST) (7) do not explicitly address their management. Consequently, clinical practice varies widely between and within departments managing musculoskeletal injuries, including trauma, orthopaedic and plastic surgeries.

### Current management

Despite the relatively low prevalence of fracture blisters quoted in the literature, management of these blisters continues to be a challenge. In general, most authors agree that early definitive fixation prior to blister formation is ideal. However, this is not always clinically feasible as the degree of soft tissue injury and resolution of tissue oedema often dictate surgical timing (8). Consequently, some surgeons may adopt a ‘watch and wait’ approach, allowing spontaneous blister resolution prior to definitive fracture fixation, a strategy that results in significant delays and prolonged inpatient stays (9). A second management strategy is active treatment of the blisters by deroofing or aspirating, followed by various topical treatments to the blister bed in an attempt to promote re-epithelialization (4, 9). Adding yet another layer of complexity to the management of fracture blisters is the previously held dogma that these blisters are sterile, a fact disproven by recent studies (10). The literature on wound healing reveals a correlation between the levels of oxygen free radicals present within the tissues and an inhibition of wound healing, demonstrating that topical administration of low molecular weight hyaluronic acid to wound sites has been shown to significantly reduce the concentration of reactive oxygen species (ROS) within the local tissue area (11, 12). These advances have gained momentum in the field of wound management within plastic surgery; however, to our knowledge, there are no such reports of this treatment being used in the context of fracture blisters, which represents an area of potential future research.

This paper aims to conduct a systematic review comprehensively assessing the existing literature regarding the pre-operative management of fracture blisters. This review intends to examine the clinical characteristics of fracture blisters, their histological features, the factors influencing their formation and the current strategies for their pre-operative management.

## Methodology

### Search strategy

Extensive electronic literature searches were performed on PubMed/MEDLINE (January 1946–May 2024), Embase (January 1974–May 2024) and Cochrane library (January 1933–May 2024). The search terms were as follows: (fracture blister OR bone blister*) AND* (*dress* OR drain* OR aspirat* OR deroof* OR manage*).

These keywords were searched in the subject headings, in title and in abstract. All reference lists of the included publications were also screened to identify any pertinent studies for cross-referencing and snowballing method. A detailed overview of the selection criteria is given in Table 1.

**Table 1:** Selection criteria.

Inclusion criteria	Exclusion criteria
English	Non-English
Full texts	Grey literature
Adult patients	Non-peer reviewed publications
Human studies	Paediatric patients
	Level of evidence below 3, including literature reviews

### Search outcome

**Figure 1:**
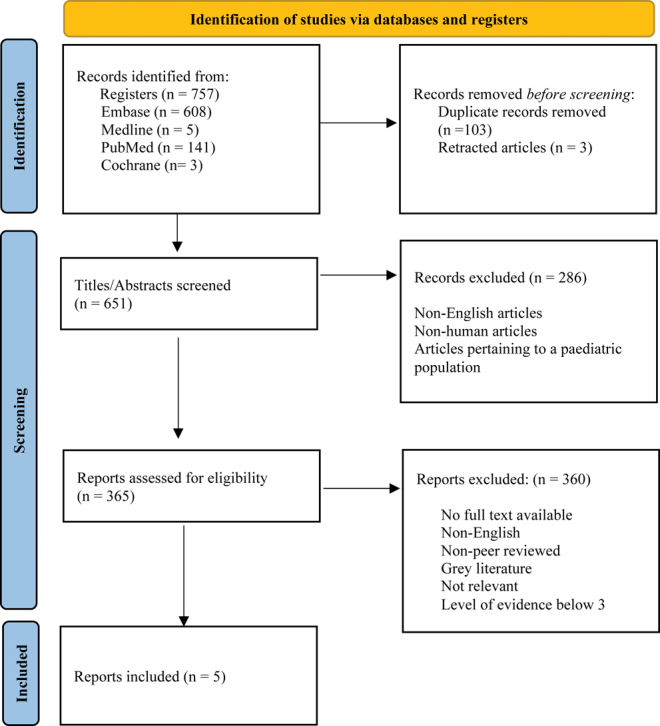
PRISMA protocol.

The review process was conducted according to the Preferred Reporting Items for Systematic Reviews and Meta-Analyses (PRISMA) (13) guidelines. All authors screened the studies for inclusion within the final review, and there were no discrepancies between inter-rater reviews. The screening and eligibility assessment processes for the search results are outlined in the PRISMA diagram (Fig. 1). All pertinent publications were tabulated, and the ROBINS-I (14) tool was utilized for risk-of-bias assessment.

## Results

The search results are tabulated in Supplementary Table 1 (see the section on Supplementary materials given at the end of the article), containing an overview of the most pertinent and highest-quality evidence available when managing fracture blisters pre-operatively. The best evidence to answer the clinical question consisted of five studies, which reported on rates of wound healing and post-operative infection, time to surgical readiness and treatment costs, following varying treatment modalities in 1162 patients.

### Risk of bias

**Figure 2:**
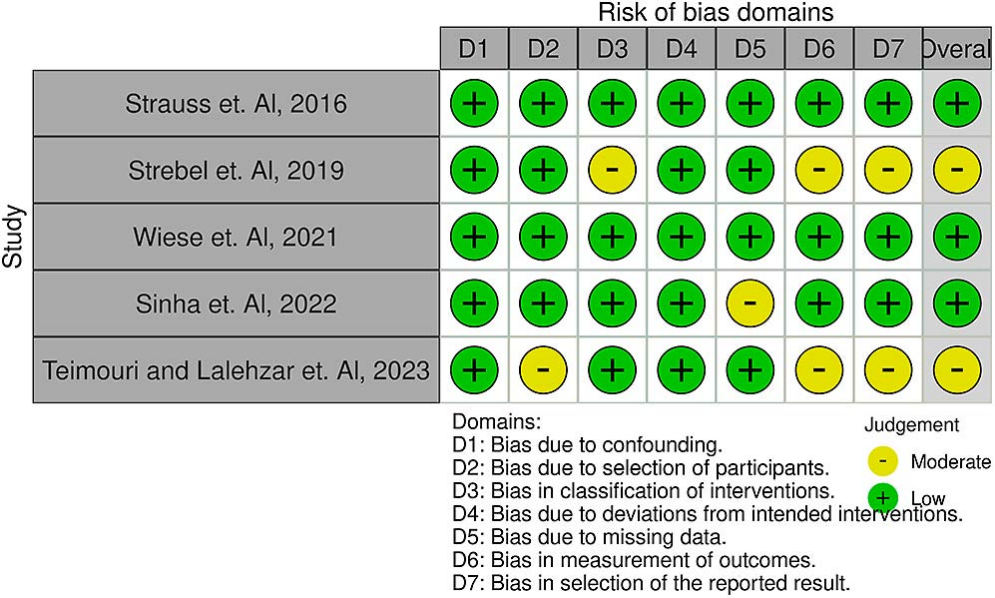
Traffic light plot depicting the risk-of-bias domains amongst included studies (10, 15, 16, 17, 18).

**Figure 3:**
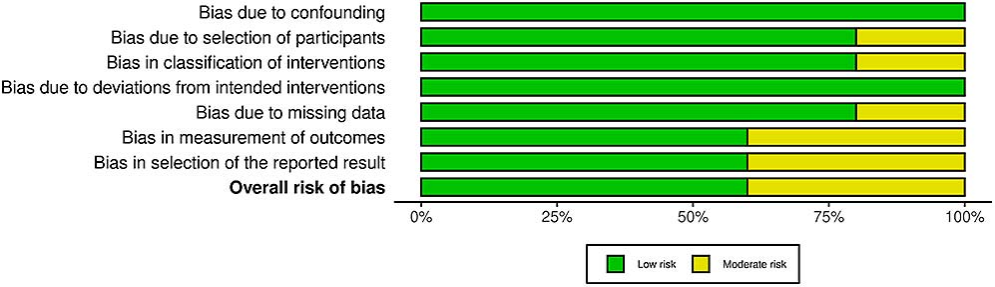
ROBINS-I tool summary plot.

The risk-of-bias assessment was conducted using the ROBINS-I tool and can be seen depicted as a traffic light plot (Fig. 2) and a summary plot (Fig. 3).

## Discussion

The aim of this systematic review was to evaluate the current pre-operative management of fracture blisters in the context of traumatic fractures. This review included five studies, which incorporated cross-sectional, prospective, retrospective and randomized controlled trial study designs. To the authors’ knowledge, this is the largest review detailing the current literature available surrounding this topic.

### Evaluation of included studies

The first paper included in our review was a prospective study described in 2006 by Strauss *et al*. (15). The authors reported on rates of fracture union, wound complications and patient satisfaction with scar appearance for 47 patients with closed, lower limb post-traumatic fracture blisters treated with deroofing and silver sulfadiazine (Silvadene, Pfizer, USA) antibiotic ointment at a level 1 trauma centre in the USA. Secondary outcomes included the delay in definitive surgical care by fracture sub-type. The authors reported soft tissue complications in 6/45 (13.3%) of the patients treated with the stipulated regimen. Four of these patients developed relatively minor post-operative wound infections: three incidents of wound breakdown and one case of superficial wound infection. However, two patients with ankle fractures and blisters within the zone of injury developed major complications, mandating re-operation. In the first instance, a diabetic patient with a haemorrhagic fracture blister developed a deep-seated bone infection post-operatively, eventually requiring an ankle fusion. The second case discussed by the authors described a patient with a clear fluid-filled blister in the zone of injury, who developed complete medial ankle skin breakdown post-operatively and returned to theatre for metalwork removal. Crucially, in both instances, wound breakdown occurred at the base of the blister bed and not at the surgical site.

Strauss *et al*. (15) included rates of fracture union as one of the primary outcomes of their study, reporting an uncomplicated fracture union rate of 95.6%. Two of the patients within the study developed non-union: one patient with a tibial plateau fracture and another with an ankle fracture. Both patients underwent revision surgery with eventual fracture union. To our knowledge, this is the only study that included this outcome as very little literature explores the link between fracture blisters and rates of fracture union. A similarly designed prospective study by Giordano *et al*. (19) conducted in 1993 quoted similar rates of post-operative wound infection (7/53, 13.2%) and an average delay in definitive surgical treatment of 7.7 days (range 0–20 days). Interestingly, the mean delay to surgery for ankle fractures was significantly less than that for both calcaneal and tibial plateau fractures (*P* = 0.02).

Silver sulfadiazine, a popular topical sulfonamide antibiotic often used in the management of burns, has grown in popularity for the management of fracture blisters and has been described by several authors (10, 15). Two randomized controlled trials relating to this topic were uncovered using the aforementioned literature search. Both studies detailed different dressing regimens for the management of the blister bed, following either aspiration or deroofing. The first study, conducted by Teimouri *et al*. (16), was a small randomized controlled trial with 31 participants. Sixteen patients were allocated to the treatment arm to receive once-weekly silver (Ag)-coated dressings, while 15 received a once-daily Gaz Vaseline dressing. The primary outcomes were healing time, patient-reported pain during dressing changes and net cost of dressing type. The authors demonstrated that the use of silver (Ag)-coated dressings had a positive effect on wound healing, cost less, was less painful during dressing changes and required less total time for dressing changes.

The second randomized controlled trial compared the outcomes of utilizing silver-impregnated fibrous hydrocolloid dressings to silver sulfadiazine cream dressings (10). The primary outcome was time to surgical readiness after complete blister re-epithelialization in both groups. Similar to the article by Teimouri *et al*. (16), the secondary outcomes included the cost associated with each treatment modality and the daily cost per inpatient hospital stay. A total of 70 patients were enrolled and completed the study protocol with 35 patients in each treatment arm. The authors reported a significant difference of four days (95% CI: 2.9–5.1 days, *P* = 0.001) in the average time to surgical readiness (SFH group, 5.3 days vs SS group, 9.3 days). No difference between the time to surgical procedure and the total length of hospital stay between the two groups was observed.

The current literature indicates that these blisters are associated with an increased risk of wound breakdown and surgical site infection when incisions are made through the blister bed, especially in blood-filled blisters (10). To appreciate the variation in the approach to the management of these blisters, Sinha *et al*. (17) reported the findings of an online questionnaire-based survey amongst orthopaedic surgeons describing their management of post-traumatic fracture blisters. The questionnaire explored surgeon-specific practices, focussing on antibiotic prophylaxis, local procedures, dressing types and additional treatment options regarding blister management. A high percentage (∼78%) of systemic, prophylactic antibiotic use was reported by the surgeons despite there being no empiric evidence or orthopaedic guidelines advocating this approach. Similarly, a large percentage of respondents (66.4%) advocated either deroofing or aspiration of blisters, with approximately 42% surgeons sending the fluid for microscopy, culture and sensitivity. More than half of the respondents used a local dressing to cover the blister bed following aspiration or deroofing.

Most studies addressing this topic have detailed fracture blister management in the context of aspirating, deroofing and variable topical dressings. Hasegawa *et al*. (20) described a novel method for fracture blister management by utilizing circumferential negative pressure wound therapy with instillation and dwell. The authors detailed two case reports whereby this new technique was trialled with success for the pre-operative management of fracture blisters in patients with underlying ankle and tibial plateau fractures. Their novel technique resulted in near-complete re-epithelialization of the decompressed blister beds within one week. In addition, no excessive surgical delay or alteration in surgical approach was necessary, with both patients eventually healing successfully with no reported post-operative wound complications. The medical devices used in these case reports are widely available and commonly used in other aspects of surgical wound care, highlighting a potential area of future research.

In 2020, Tosounidis *et al*. (4) described fracture blister pathophysiology and management in addition to providing a review on the current literature. Their article included publications on the subject from 1993 to 2006, summarizing the current management strategies. Four years after this study, two randomized controlled trials have been conducted, adding to the existing literature on the management of these blisters. The authors recommended a preventative approach whereby limb elevation, cryotherapy, ice therapy and intermittent pneumatic compression were used as techniques to prevent fracture blister formation in the first instance. If blisters were to develop, a staged or delayed approach utilizing external fixator devices as a bridge to definitive fracture fixation was recommended. The authors also concluded that blister aspiration and/or deroofing has not been substantiated by the literature and advised that fracture blisters should be left intact and covered with soft dressings until the time of definitive fracture fixation. The promising findings from the randomized controlled trial conducted by Wiese *et al*. (18) in 2021, however, suggest that blister deroofing and silver-impregnated fibrous hydrocolloid dressing application to the blister bed may result in faster wound re-epithelialization and time to surgical readiness.

### Strengths of our study

Within this systematic review, the search terms and strategy were specific and comprehensive, ultimately yielding detailed and pertinent results reflecting the current global practices for the peri-operative management of fracture blisters. Numerous databases and registers were utilized, and each source was subjected to risk assessment analysis using the ROBINS-I tool. Screening of the material was undertaken by multiple authors utilizing PRISMA guidelines, with studies deemed to be of poor quality ultimately being excluded from this review. The results of this systematic review include the findings of two recent randomized controlled trials with evidence in support of blister aspiration/deroofing and subsequent treatment of blister beds. The included studies also highlight the fact that fracture blisters are not sterile and should not be deroofed intra-operatively.

### Limitations of our study

The limitations of this review include the relative paucity of high-quality evidence available on the topic. The cross-sectional and prospective study designs in addition to the risk of volunteer response bias from the survey-based studies must be noted. In addition, the studies that utilized survey-based methodologies were created *de novo* and were not subjected to external validity and reliability testing. A final limitation of this review is the fact that the authors were not able to undertake a meta-analysis, given the heterogeneity and scarcity of pertinent studies.

## Conclusion

Post-traumatic fracture blisters remain a clinical dilemma. They are associated with a delay in time to surgery, suboptimal surgical approaches and post-operative wound complications. Currently, no uniform consensus regarding the optimal management of fracture blisters exists. The findings from this review challenge the previously held dogma that these blisters are sterile, re-emphasizing that if present within the zone of injury, they should not be deroofed intra-operatively and skin incisions should not be made through them. Following deroofing, re-epithelialization occurs sooner in serous blisters compared to haemorrhagic ones and the time to surgical readiness can be improved if the blister bed is treated with a topical agent. Recent studies have reported favourable outcomes utilizing silver sulfadiazine antibiotic formulations for blister bed treatment, while one randomized controlled trial reported superior rates of time to surgical readiness with silver-impregnated fibrous hydrocolloid dressings. Further research with high-quality prospective randomized trials is advisable in order to confirm the best clinical management of fracture blisters while accounting for stratification and selection bias.

## Supplementary materials



## ICMJE Statement of Interest

The authors declare that there is no conflict of interest that could be perceived as prejudicing the impartiality of the work.

## Funding Statement

This work did not receive any specific grant from any funding agency in the public, commercial or not-for-profit sector.

## Author contribution statement

IR: conceptualization (lead); writing – original draft (lead); formal analysis (lead); writing – review and editing (equal). KS: methodology (lead); formal analysis, review and editing (equal), writing – review and editing (equal). AD: writing – review and editing (equal). AC: conceptualization (supporting); writing – original draft (supporting); Writing – review and editing (equal).
