# Supplementary material for: Pre-operative management of fracture blisters: a systematic review
Source: EFORT Open Rev. 2025 Mar 3;10(3):e20240074. doi: 10.1530/EOR-2024-0074 (PMC11896682; doi:10.1530/EOR-2024-0074)
Supplement: Supplementary file 1 [file supplementary_materials.pdf]

Supplementary Table 1: Search results (all percentages have been rounded to the nearest integer)

**Paper title, Author, journal, country, date, Study type and level of evidence**	**Patient/Study groups and treatments**	**Outcomes**	**Key results**	**Comments**
Blisters Associated With Lower-Extremity Fracture: Results of a Prospective Treatment Protocol<br><br>Strauss et al[1]<br><br>Journal of Orthopaedic Trauma<br><br>USA<br><br>2006<br><br>Prospective study,<br>Level II	655 patients over 4-year study period<br><br>*Inclusion criteria:* Closed, lower limb fractures with blisters in the zone of injury.<br><br>*Patients with fracture blisters* = 47/655; 7.2%<br>- Single blister = 28<br>- Multiple blisters = 19<br><br>*Blister characteristics:*<br>- Clear-filled = 22<br>- Blood-filled = 20<br>- Combination = 5<br><br>*Fracture patterns:*<br>- Ankle = 17<br>(OTA 44)<br>- Tibial plateau = 13 (OTA 41)<br>- Tibial shaft = 5<br>(OTA 42)<br>- Calcaneus = 8 (OTA 45)<br>- Pilon = 4<br>(OTA 43)<br><br>*Treatment:*<br>Blisters unroofed with silver sulfadiazine	Fracture union<br><br>Development of wound or skin complications<br><br>Patient satisfaction with cosmetic outcome (assessed through telephone surveys)<br><br>Delay in definitive surgical care, mean, (range of days)<br><br>Residual pain in the region of the previous fracture was reported on a scale of 1 to 10 (no pain to severe pain) with a mean of 1.7 (range 0 to 7)	Fracture union 43/45 (96%)<br><br>Soft tissue complication rates 6/45 (13%)<br><br>Uncomplicated post-operative course at 27 weeks (range 14 to 35)<br>37/45 (82%)<br><br>Scarring (at 51.3 months follow up) = 21%<br><br>$\bar{X}$= 7.7 days, (0-20 days)<br><br>Ankle = 6 (0 to 18 days)<br>Tibial plateau = 11 days, (0 to 20 days, $p < 0.02$)<br>Calcaneus = 12 (4 to 19 days, $p < 0.02$)	Missing data for delay in definitive surgical care for tibial shaft and pilon fractures<br><br>*Follow-up:*<br>- 2/47 patients lost to follow-up<br>- 14 patients were unreachable for the follow-up portion<br>of the investigation<br><br>*2 major complications:* - - Deep wound infection following ankle ORIF, mandating ankle fusion<br>- Medial skin breakdown following ankle fracture necessitating metal work removal

	(Silvadene) antibiotic cream applied twice daily			
A retrospective analysis of the aspiration of fracture blisters<br><br>Strebel et al[2]<br><br>Journal of Clinical Orthopaedics and Trauma<br><br>USA<br><br>2019<br><br>Retrospective study, Level III	N = 64<br><br>*Types:*<br>- Blood-filled = 29<br>- Serous = 35<br><br>*Location:*<br>- Leg = 15 (23%)<br>- Ankle = 33 (52%)<br>- Foot = 13 (20%)<br>- Upper extremity = 3 (5%)<br><br>*Treatment:*<br>Aspiration of blisters alone	Rates of post-operative wound infection<br><br>Culture of fluid aspirate	Post-operative infection = 4/64 (6%)<br><br>*Colonisation:*<br>- Sterile = 57 (89%)<br>- Colonised = 7 (11%)	None of the infections were in patients with a colonised aspirate.<br><br>The only predicting factor of infection risk identified was the placement of skin incision through or near haemorrhagic blisters
Randomized Controlled Trial Comparing Silver-Impregnated Fibrous Hydrocolloid Dressings With Silver Sulfadiazine Cream Dressings for the Treatment of Fracture Blisters to Determine Time to Surgical Readiness	N = 70<br><br>*Treatment:*<br>Silver-impregnated Fibrous Hydrocolloid Dressings SFH (n = 35; 50%)<br><br>Silver Sulfadiazine cream dressings (n = 35; 50%)	*Time (days) to:*<br>- Surgical readiness<br><br>- Surgical procedure<br><br>- Discharge (length of stay)	SFH = 5.3 +/- 2.6<br>SS = 9.3 +/- 2.0<br>MD = 4.0<br>p<0.001<br><br>SFH= 10.5 +/- 5.5<br>SS= 11.6 +/- 2.9<br>MD= 1.2<br>p=0.268<br><br>SFH= 12.3 +/- 6.4<br>SS=12.8 +/- 3.0<br>MD= 0.5<br>p= 0.688	Zero patients lost to follow-up<br><br>The principal investigator held the responsibility for administering the treatment and conducting daily assessments, introducing a potential for bias in the evaluation of blister re-epithelialization.

Wiese et al[3]<br><br>Journal of Orthopaedic Trauma<br><br>South Africa<br><br>2021<br><br>Randomised controlled trial, Level I		*Treatment costs (USD):*<br><br>- Dressing cost based on the mean time<br>to re-epithelialization<br><br>- Hospital stay cost based on the mean<br>time to surgical readiness<br><br>- Total treatment cost until surgical<br>readiness	 <br>SFH =$4.77<br>SS = $6.79<br><br>SFH = $370.47<br>SS = $650.07<br><br>SS = $375.24<br>SFD = $656.86	
The Current Consensus on the Management of Post-traumatic Blisters Among Orthopaedic Surgeons<br><br>Sinha et al[4]<br><br>Indian Journal of Orthopaedics<br><br>2022<br><br>Prospective question-based survey, Level II	N = 342	Administration of systemic antibiotics = (n =267; 78%)<br><br>Deroofing/aspiration of blisters (n =227; 66%)	*Antibiotic use:*<br>(i) for all patients developing blisters n = 171; 50%)<br>(ii) In haemorrhagic blisters (n = 75; 22%)<br>(iii) In serous blisters<br>(n = 21; 6%)<br>(iv) Not at all (n =75; 22%)<br><br>*Management strategy:*<br>- Allow blisters to heal on their own (n = 115; 34%)<br>- Aspiration of blisters (n =149; 44%)<br>- Deroofing of blisters (n =78; 23%)<br><br>*Topical dressings:*<br>- Yes (n =209; 61%)<br>- No (n =133; 39%)	Survey covers common blister management options but is not fully representative of all strategies<br><br>Missing data from hygroscopic dressing category, leaving reader to calculate results on own

			Dressing types:<br>- Betadine (n =53; 25%)<br>- Normal saline n = 78; 37%)<br>- Paraffin gauge (n =65; 31%)<br>Hygroscopic dressing (n = 13; 6%)	
Evaluation of the therapeutic effect of dressing containing Silver (Ag-coat) in the process of healing skin blisters caused by limb fractures: a clinical trial study<br><br>Teimouri and Lalehzar[5]<br><br>BMC Surgery<br><br>Iran<br><br>2023<br><br>Randomised controlled trial,	N = 31 patients with lower limb post-traumatic fracture blisters<br><br>*Types of dressings:*<br>- Silver-coated once weekly changes (n=16; 52%)<br>- Gaz Vaseline daily changes (n=15; 48%)<br><br>*Assessments:*<br>- Wound healing, assessed on days 1,7 and 14<br>- Duration of visit for dressing changes (time, minutes)<br><br>*Other measures:*<br>- Number of dressings<br>- Net cost of dressings<br>- Pain during dressing changes (Visual	Duration of visit (min/day)	- Ag coat= 16.68 ± 4.19<br>- Gaz Vaseline = 27 ± 7.02	Small study (n = 31)<br><br>Patients lost to follow up before re-evaluation of wound healing at day 7 and 14<br><br>Study design not entirely clear.<br><br>From the Method and Materials section, it appears that the blisters were drained intra-operatively prior to application of the respective dressing types
		Number of dressings (mean ± SD)	- Ag coat= 3 ± 0.00<br>- Gaz Vaseline = 43.46 ± 14.15	
		Net cost of dressing (Iranian Rial [ریال], British Pound [£])	- Ag coat = ریال 2,425,361.25 ± 938118.75 (£45.05 ± 17.43)<br>- Gaz Vaseline = ریال 8,693,333 ± 2830312.11<br>(£161.49 ± 52.58)	
		Pain assessment - VAS Score (Visual Analogue Scale)	*Ag coat*<br>- Day 0 = 5.34 ±0.78<br>- Day 7 = 3.96 ±0.80<br>- Day 14 = 2.56 ±0.60<br><br>*Gaz Vaseline*<br>- Day 0 = 7.70 ±0.88<br>- Day 7 = 6.50 ±0.96	

Level II	Assessment Score – VAS)	 <br><br><br><br><br>Wound area (cm^2)	- Day 14 = 5.13 ±0.93<br><br><br><br><br>*Ag coat*<br>- Day 0 = 23.43 ± 9.93 - Day 7 = 20.65 ± 9.61 - Day 14 = 17.01 ± 9.18<br><br>*Gaz Vaseline*<br>- Day 0 = 27.28 ± 10.89 - Day 7 = 26.88 ± 10.86 - Day 14 = 26.44 ± 10.82	
	**N = 1162**			

Key: *Ag – Silver; OTA – Orthopaedic Trauma Association fracture classification; SD – standard deviation; SFH – Silver-impregnated Fibrous Hydrocolloid; SS - Silver Sulfadiazine; MD – Mean Difference; Iranian Rial* [ریال]; *USD – United States Dollars;* $\bar{X}$ *- Mean*

1. Strauss EJ, Petrucelli G, Bong M, Koval KJ, Egol KA. Blisters associated with lower-extremity fracture: Results of a prospective treatment protocol. *Journal of Orthopaedic Trauma.* 2006 Oct;20(9):618–22. doi:10.1097/01.bot.0000249420.30736.91

2. Strebel SJ, Burbank KM, Tullar JM, Jenkins MD, Caroom C. A retrospective analysis of the aspiration of fracture blisters. *Journal of Clinical Orthopaedics and Trauma.* 2020 Feb;11. doi:10.1016/j.jcot.2019.11.002

3. Wiese KR, van Heukelum M, Lombard CJ, Ferreira N, Burger MC. Randomized controlled trial comparing silver-impregnated fibrous hydrocolloid dressings with silver sulfadiazine cream dressings for the treatment of fracture blisters to determine time to surgical readiness. *Journal of Orthopaedic Trauma*. 2021 Aug;35(8):442–7. doi:10.1097/bot.0000000000002042

4. Sinha S, Kumar A, Jameel J, Qureshi OA, Majeed A, Kumar S. The current consensus on the management of post-traumatic blisters among orthopaedic surgeons. *Indian Journal of Orthopaedics.* 2022 Feb 28;56(6):1011–7. doi:10.1007/s43465-022-006129

5. Teimouri M, Lalehzar S sadat. Evaluation of the therapeutic effect of dressing containing silver (ag coat) in the process of healing skin blisters caused by limb fractures: A clinical trial study. *BMC Surgery.* 2023 Apr 28;23(1). doi:10.1186/s12893-023-02012-8
